# The Physical Vapor Transport Method for Bulk AlN Crystal Growth

**DOI:** 10.3390/molecules24081562

**Published:** 2019-04-19

**Authors:** Wen-Hao Chen, Zuo-Yan Qin, Xu-Yong Tian, Xu-Hui Zhong, Zhen-Hua Sun, Bai-Kui Li, Rui-Sheng Zheng, Yuan Guo, Hong-Lei Wu

**Affiliations:** Key Laboratory of Optoelectronic Devices and Systems of Ministry of Education and Guangdong Province, College of Optoelectronic Engineering, Shenzhen University, Shenzhen 518060, China; chenwenhao2017@email.szu.edu.cn (W.-H.C.); 2176285305@email.szu.edu.cn (Z.-Y.Q.); tian365xy@163.com (X.-Y.T.); 2160190108@email.szu.edu.cn (X.-H.Z.); szh@szu.edu.cn (Z.-H.S.); libk@szu.edu.cn (B.-K.L.); rszheng@szu.edu.cn (R.-S.Z.); guoyuanlg@sina.com (Y.G.)

**Keywords:** bulk AlN crystal, inverse temperature gradient, dominant growth, crystalline quality, physical vapor transport

## Abstract

In this report, the development of physical vapor transport (PVT) methods for bulk aluminum nitride (AlN) crystal growth is reviewed. Three modified PVT methods with different features including selected growth at a conical zone, freestanding growth on a perforated sheet, and nucleation control with an inverse temperature gradient are discussed and compared in terms of the size and quality of the bulk AlN crystals they can produce as well as the process complexity. The PVT method with an inverse temperature gradient is able to significantly reduce the nucleation rate and realize the dominant growth of only one bulk AlN single crystal, and thus grow centimeter-sized bulk AlN single crystals. X-ray rocking curve (XRC) and Raman spectroscopy measurements showed a high crystalline quality of the prepared AlN crystals. The inverse temperature gradient provides an efficient and relatively low-cost method for the preparation of large-sized and high-quality AlN seed crystals used for seeded growth, devoted to the diameter enlargement and quality improvement of bulk AlN single crystals.

## 1. Introduction

In recent years, aluminum nitride (AlN) has attracted increasing attention in the field of electronics and optoelectronics due to its excellent optical, electrical, mechanical, and piezoelectric properties. Its high breakdown field strength, high thermal conductivity, high carrier saturation velocity and high radiation tolerance offer extensive application potential in high temperature-resistance, high-frequency, anti-radiation, and high-power electronic devices [1,2]. At present, AlN is considered the most promising substrate material for III-nitride semiconductor devices [3,4]. In contrast with substrates such as sapphire and silicon carbide (SiC), AlN substrates provide more lattice and a thermal expansion match with gallium nitride (GaN) and aluminum gallium nitride (AlGaN), leading to a low dislocation density (DD) in over-grown active layers. A number of studies have confirmed that using AlN as buffer layers can greatly improve the quality of GaN [5,6]. Furthermore, AlN possesses an extra wide direct bandgap of 6.2 eV, which presents great potential for application in deep-ultraviolet (DUV) optoelectronic devices such as light emitting diodes (LEDs), laser diodes (LDs) and photodetectors. These devices have very wide application prospects in air disinfection, water purification, ultraviolet curing, fire detection and other relevant technologies. Yoshitaka Taniyasu et al. reported an AlN-based LED with an emission wavelength of 210 nm in 2006 [7], which greatly promoted the development of solid-state DUV light sources. Wei Zheng et al., reported a vacuum-ultraviolet (VUV)-sensitive (λ < 200 nm) photodetector based on high-quality AlN nanowires [8]. To realize further performance improvement of DUV or VUV optoelectronic devices, large-sized and high-quality bulk AlN single crystals and reliable preparation methods are necessary.

AlN is a synthetic mineral that doesn’t exist naturally. In the past six decades, different methods have been developed to prepare bulk AlN single crystals, including direct-nitridation of aluminum, hydride vapor phase epitaxy (HVPE) [9] and physical vapor transport (PVT) [10,11,12,13]. The AlN single crystal with a diameter of 0.03 mm was first synthesized in 1956 [14]. In 1976, bulk AlN crystals, 10mm in thickness and 3 mm in diameter, were prepared by the sublimation-recondensation method [10], which laid the foundation for the PVT growth of bulk AlN crystals. PVT is the most suitable and widely used method for bulk AlN crystal growth. The largest AlN wafer prepared by PVT is approximately up to 2-inches in diameter so far [15,16]. Nowadays the diameter enlargement of AlN single crystals is the most crucial requirement. On the other hand, the size and structural properties of grown AlN crystals are significantly affected by growth conditions such as temperature, ambient pressure, crucible material and the type of seed. This work investigates different modified PVT growth strategies and particularly analyzes the attributes of the inverse temperature-gradient method in terms of the size and crystal quality of the grown AlN crystals.

## 2. Mechanism

The growth process of AlN crystals by PVT method is summarized in the following three steps: (I)In a nitrogen atmosphere, the AlN powder sublimate in a closed or semi-closed crucible with certain temperature and pressure, producing gaseous Al and N_2_; (II)The vapor species transfer along a temperature gradient from the higher-temperature source zone to the lower-temperature crystalline zone; (III)The collision, diffusion, absorption and desorption of vapor species on the substrate promote the crystallization of AlN. The sublimation and recondensation process can be briefly described by the following formula

(1) $$2\mathrm{AlN}\left(s\right)⇌2\mathrm{Al}\left(g\right)\;+\;N_{2}\left(g\right)$$

Other than the main species (gaseous Al and N_2_), Al_n_N(g)(n = 2,3,4) possibly exists in spite of its much low concentration. Al_3_N presents an obvious advance in mole fraction and growth rate among Al_n_N vapor species [17]. It might play an important role in the deposition of AlN because the higher pressure of nitrogen leads to a lower concentration of Al_3_N, which is related to the decrease of the growth rate [18]. However, further influences of Al_n_N(g)(n = 2,3,4) on AlN crystal growth has not been determined in the experiment.

The growth rate of AlN crystals is sensitive to the mass transport of gaseous Al and N_2_, both of which are the main gas-phase species in the PVT growth system. There have been different models developed to describe the impact of vapor phase transports on growth rate [19,20,21,22,23,24]. The AlN crystals are grown in a nitrogen environment. With a high ambient pressure (~a nitrogen atmosphere), the vapor phase transport is dominated by the diffusion mechanism. But in a vacuum (≈10^−4^Torr), the drift mechanism of transport plays a predominant role [19]. The proper ambient pressure needs to be considered in terms of growth rate. Under an excessively high ambient pressure, the sublimation rate of the source material would be sharply limited.

According to the models based on vapor diffusion (P_tot_~600Torr) [20,21], in a usual N-rich PVT growth system where partial pressure of nitrogen is considerably higher than that of gaseous Al, the growth rate is largely limited by the supply of Al species to the growth surface at temperatures above 2000 °C. Considering the surface kinetics, the adsorption behavior of N_2_ on the growth surface will also limit the growth rate due to the high dissociation energy of N_2_, especially at Al-rich conditions [22]. The sticking coefficient of N_2_, an important factor in the adsorption process, can be increased at higher temperature. High growth temperature and nitrogen pressure contribute to the adsorption of N_2_ on the crystalline surface, and thus comparatively increase the growth rate. Under the N-rich condition the growth rate limitation caused by surface kinetics mechanism is insignificant, but the suppression of N_2_ adsorption cannot be ignored at a nearly stoichiometric vapor phase [19,23,24]. Therefore, the determinant factor influencing the growth rate will be either the gaseous Al transport or the N_2_ adsorption on the AlN surface, depending on the specific growth conditions.

In order to prepare bulk AlN crystals with a sufficiently low nucleation rate and a high enough growth rate (>100 μm/h) by PVT method, adequate supersaturation should be realized. Based on the thermodynamic properties of species in the Equation (1), the supersaturation value at the growing region is defined as [13,23]

(2) $$S=\frac{{\left(p_{Al}\right)}^{2}\cdot \left(p_{N_{2}}\right)}{K\left(T\right)}-1$$

where p_Al_ is the vapor pressure of Al, p_N2_ the vapor pressure of N_2_, and K(T) the equilibrium constant. The supersaturation value reflects the crystallization rate at the growing surface. The equilibrium is reached between crystallization and decomposition when S = 0. The crystallization rate increases with the supersaturation value when S > 0. For AlN growth by spontaneous nucleation, an excessively low local supersaturation will suppress the nucleation and lead to a low growth rate, whereas an excessively high local supersaturation will result in a high nucleation density or even polycrystallization. The neighboring grains are detrimental to both diameter enlargement and structural quality perfection of growing AlN single crystals.

**Figure 1:**
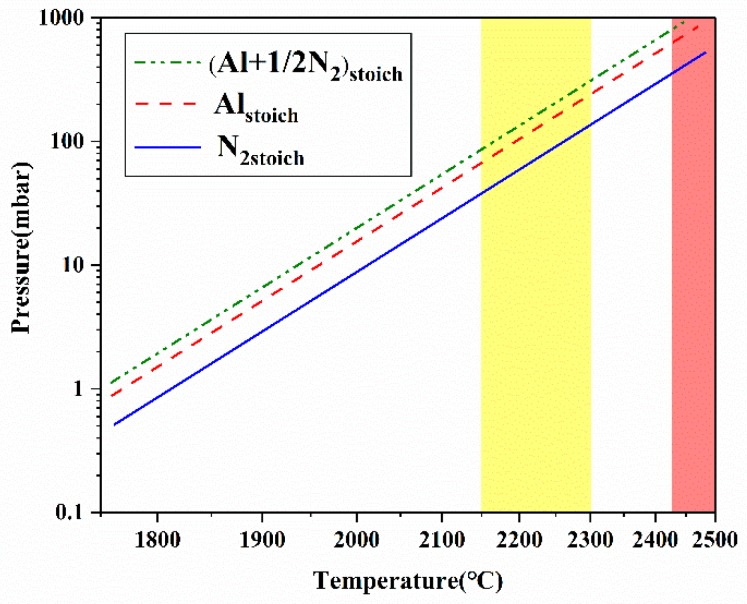
Partial pressure of Al and N_2_ versus temperature in the AlN-N_2_ system [25]. The yellow colored area highlights the suitable temperature window for growth of bulk AlN crystals. The red colored area reflects the limitation of the AlN decomposition point.

Figure 1 shows the rising trends of the vapor pressure of major species with increasing temperature in the AlN-N_2_ system [25]. The significant increase of Al vapor pressure largely contributes to the supersaturation increase. Note that the temperature difference between the source and crystalline zone (ΔT) motivates the species to transport to the growth surface. Therefore, with a considerable excess of N_2_ species in the growth system that has a dominant diffusion mechanism, the supersaturation and growth rate climb with the increasing growth temperature (T_G_) and especially the increasing ΔT. A proper axial temperature gradient plays a key role in the control of supersaturation. According to Equation (2) and numerical simulations of the thermal field, the supersaturation value at the range of 0.25~0.3 [26] is adequate for both growing bulk AlN single crystals with growth rates of above 100 μm/h and realizing low nucleation densities. Furthermore, the defect generation such as dislocation and small angle grain boundary (LAGB) during growth is significantly influenced by the temperature gradient. Except for the axial temperature gradient, an appropriate radial temperature gradient around the growing AlN crystal can also improve the crystal quality by suppressing the polycrystalline nucleation and reducing the thermal-elastic stresses in crystals [27].

In a nitrogen atmosphere, with temperatures over 2150 °C, the growth anisotropy (partial to c-direction) of wurtzite AlN is weakened, promoting the diameter enlargement of bulk AlN single crystals. It is worth mentioning that the upper growth temperature is limited by the decomposition point at around 2430 °C with a nitrogen pressure of 1 bar [10], as shown in the red colored area in Figure 1. Upon reaching the decomposition temperature, AlN decomposes into gaseous species. A lot of generated gaseous Al and even liquid Al can cause corrosion to the crucible (e.g., tungsten crucible) and decrease its lifetime. In view of the adequate supersaturation, the endurance of crucible and the demand of large-sized AlN crystals with high structural quality, the growth temperature and axial temperature gradient should be appropriately controlled at the range of 2150~2300 °C and 5~20 °C/mm in a nitrogen atmosphere. Correspondingly the suitable growth window for AlN bulk growth is highlighted with a yellow color in Figure 1.

## 3. Different PVT Methods

The growth of AlN crystals is considerably influenced by the temperature distribution and growing anisotropy, which make it difficult to achieve further diameter enlargement of bulk AlN single crystals. AlN-seeded growth is the best way to realize further enlargement of diameter and improvement of crystalline perfection of AlN crystals. However, large-scale and high-quality AlN seeds are difficult to obtain, which only can be acquired from the previously grown AlN crystals. Self-seeding growth is still playing an indispensable role in AlN bulk growth. At present, there are three modified PVT methods for AlN growth by spontaneous nucleation: (i) selected growth at a conical zone, (ii) freestanding growth on a perforated sheet, and (iii) nucleation control with an inverse temperature gradient. Mastering the efficient and low-cost method to prepare acceptable AlN seed crystals is necessary for seeded growth. The inverse temperature-gradient method is designed for our PVT growth experiments.

### 3.1. Selected Growth at Conical Zone

**Figure 2:**
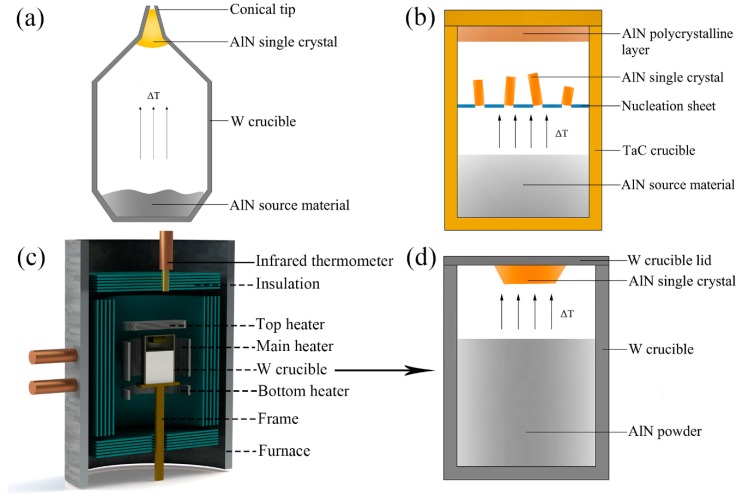
Schematic diagrams of three different growth setups: (**a**) A tungsten crucible with a conical tip designed for selected growth [28]. (**b**) A TaC crucible with a perforated sheet designed for separate freestanding growth [26]. (**c**) A growth unit with three heated zones. (**d**) A tungsten crucible deposited in setup (**c**) for the inverse temperature-gradient method.

Owing to the influence of anisotropic growth on AlN crystal enlargement, the AlN single crystals grown on a planar crucible lid generally sustain the stress from surrounding crystallites in the nucleation region, which limits the further expansion of crystal size and generates structural defects. In view of the adverse impacts between neighboring AlN crystals during growth, a conical zone was designed as the nucleation region in a tungsten (W) crucible [28], as shown in Figure 2a. The tip of the conical crucible allows a single crystal to grow in a dominant position. Additionally, the tip filled with an AlN single crystal can be cut off and then employed as a seed portion in seeded growth to achieve diameter enlargement. Using this technology, AlN wafers up to 2-inch in diameter with a usable area of ~85% and a low average etch pit density (EPD < 10^4^ cm^−2^) were obtained [29].

### 3.2. Separate Freestanding Growth on Perforated Sheet

For low-density nucleation and crystallization, a perforated sheet is designed as a nucleation area between the bottom source zone and crucible lid in the TaC crucible [26], as shown in Figure 2b. During growth, the nucleation and crystallization proceed on both the perforated sheet and the crucible lid, producing separate freestanding AlN single crystals and a polycrystalline AlN layer, respectively. The local supersaturation at nucleation area is controlled in a suitable range to obtain low nucleation density and large-sized unstressed single AlN crystals with a growth rate of up to 200 μm/h. Bulk AlN single crystals of 9 × 9 × 14 mm^3^ were prepared by this setup, which have high structural quality with low dislocation densities (DD ≤ 10^4^ cm^−2^) and no LAGBs [13,26].

### 3.3. Inverse Temperature-Gradient Growth on Crucible Lid

For AlN bulk growth by spontaneous nucleation, further improvement of size and crystalline quality cannot be achieved without nucleation control. A proper temperature field is necessary to control the supersaturation at nucleation area and acquire a sufficiently low nucleation rate. As mentioned above, temperature difference is a major impetus for vapor transport and crystal deposition. Therefore, an inverse temperature gradient can be used for nucleation control [12]. A three-zone heated furnace, including three heaters (main/top/bottom heater) and their respective infrared thermometers for temperature measurement, has been designed to prepare bulk AlN single crystals, as shown in Figure 2c. The AlN powder as source material is preliminarily sintered to remove the oxygen impurity and the purity of it can be above 99.9%. In such a growth setup, as shown in Figure 2d, a vertical thermal gradient is established in a W crucible and AlN is deposited on the crucible lid where thermal equilibrium forms. Bulk AlN single crystals are prepared at a growth temperature of 2250 °C with a growth rate >200 μm/h by this method. The typical crystals prepared through this technique are evaluated in the next section. During the experiment process with a high purity nitrogen atmosphere (99.999%) of 800Torr, the regulation of temperature in crystalline zone (Tc) and source zone (Ts) can be mainly divided into three steps:

(1) During heating, Tc > Ts, the inverse temperature gradient (ΔT < 0) is established to suppress the nucleation at relatively low growth temperature (<2150 °C).

(2) During the holding period, Tc < Ts, the positive temperature gradient (ΔT > 0) promotes the growth of AlN single crystals on the nucleation area with proper ΔT of 5~20 °C/mm.

(3) During cooling, before the temperature of source zone falls below 1750 °C, at which time the source AlN material stops subliming, an inverse temperature gradient is maintained to suppress the recrystallization on the surfaces of AlN crystals.

## 4. Results and Discussion

### 4.1. Method Comparison

Three modified PVT methods for AlN bulk growth all present prominent effects on the control of nucleation rate and the comparison of them is summarized in Table 1. Both of the other two strategies aim at reducing the nucleation rate by adjusting the environment of nucleation, which is realized by changing the shape of crucible or setting up a growth site with suitable supersaturation. For inverse temperature-gradient method, the nucleation can be effectively controlled by the temperature gradient.

Compared with the spontaneous nucleation on a planar crucible lid, the dominant single crystal at the conical tip is more capable of expanding into a large-sized single crystal. However, it is hard to guarantee the growth of only one single crystal in the conical portion. Furthermore, such a crucible needs a high enough joint quality between the conical portion and the source portion due to its special construction, leading to a high process complexity. Both precise cutting of the conical tip and separation of the AlN single crystal and the tip will also increase the production cost.

The structural design of separate freestanding growth aims at creating a proper single-crystal growth environment by regulating the distributions of temperature and supersaturation. In particular, the c-facet wafers cut from the freestanding AlN crystals contain a large N-polar (00$\overset{-}{1}$) facet, which presents a step-flow growth mode [30]. Compared with the Al-polar growth direction, the N-polar growth direction is preferable in favor of diameter enlargement and superior structural quality [30,31]. The c-facet wafers from (00$\overset{-}{1}$) grown AlN crystals with a low defect density are suitable as seeds for further N-polar seeded growth. The carbon impurities from the TaC crucible can be pumped out by forming CO with the residual oxygen to reduce the threat to the structural quality of AlN crystals. At the cooling stage of growth, the large difference of thermal expansion coefficient between TaC and AlN may produce compressive stress on AlN crystals and lead to cracks and defects. Furthermore, there probably exists interfacial reactions between TaC and vapor Al, producing Al_4_C_3_ and TaAl_3_ [32]. In fact, the major problem is the manufacture process because the machining of sintered TaC crucibles [33] is so complicated that the manufacturing cost is higher than other frequently-used crucibles like W crucibles.

**Figure 3:**
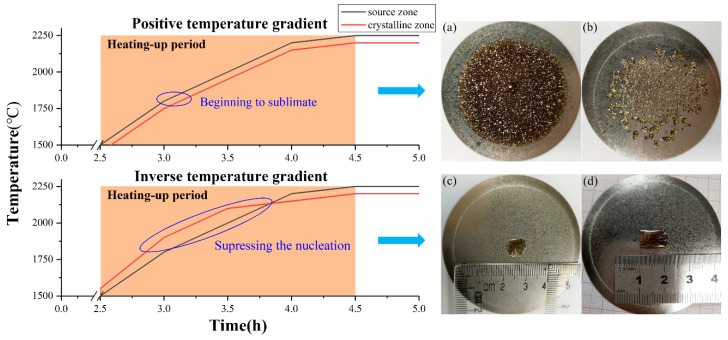
The temperature control process of heating with a positive temperature gradient (ΔT > 0) and an inverse temperature gradient (ΔT < 0). Images (**a**,**b**) and (**c**,**d**) on the right side respectively show the crystallization of AlN without and with inverse temperature-gradient method.

The biggest advantage of inverse temperature-gradient method is the dominant growth of only one bulk AlN single crystal on the W substrate during one growth period. Under a nitrogen pressure of 1 bar, the AlN powder begins to sublimate when the temperature reaches around 1800 °C, so heating-up stage is the critical period for nucleation control. Figure 3 shows the temperature control process in the positive temperature gradient (ΔT > 0) and the inverse temperature gradient (ΔT < 0), respectively. During heating, a positive temperature gradient easily led to a lot of nucleation points, which caused polycrystallization as shown in Figure 3a,b. By establishing an inverse temperature gradient in the heating stage, the nucleation could be suppressed, and as a result, the nucleation density was remarkably reduced. As shown in Figure 3c,d, only one centimeter-sized AlN single crystal grown by spontaneous nucleation can be clearly observed on the center of the crucible lid. With a single dominant growth mode, the center AlN single crystals can avoid being affected by the neighboring grains, and thus realize the diameter enlargement and the reduction of defects. Besides, the orientation of AlN crystal growth with either c- or a-orientation can be controlled by a thermal field in this setup. At growth temperatures of 2200~2250 °C, a relatively low ΔT (approaching the radial temperature difference on the substrate) can promote the lateral transfer of gas-phase species on growth surfaces and contribute to the growth of nonpolar plane (like a-facet) AlN crystals despite of a low growth rate. As such, both polar and nonpolar plane used for specific-surface seeded growth can be obtained with a large size by using this method. More details were shown in ref [12]. The most vital factor of the inverse temperature-gradient method is the accuracy of the temperature control, which is the major reason why the furnace has three infrared thermometers for different zones.

In addition, the most used crucible materials are W and TaC at present. The W crucible used for the inverse temperature-gradient method is assembled by a rounded lid and a cylindrical body, which can be recycled in a W/Molybdenum (Mo)- based unit. Compared with the conical structure and high cost sintering of TaC crucible, the straight W crucible largely reduces the production cost and offers a higher operational flexibility for the method. Considering that W will react with C to form WC at temperatures of 1400~1800 °C [34], graphite materials should be forbidden in this growth unit. The use of W material avoids the carbon impurities that are harmful to the quality of the AlN crystals and W hardly exists in the bulk AlN crystals.

According to the above comparison and analysis, the inverse temperature-gradient method applied with the straight W crucible presents a more obvious effect on the nucleation control and provides a strategy with a lower cost for production of AlN seed crystals.

### 4.2. Characterization

**Figure 4:**
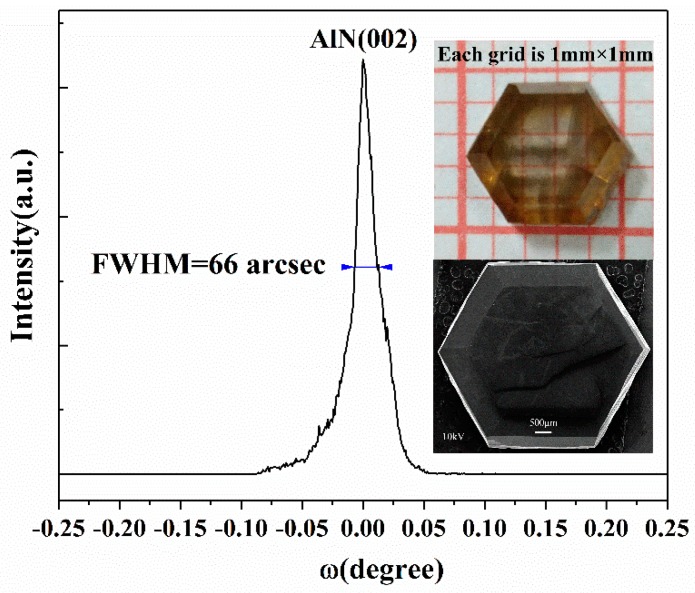
The X-ray rocking curve of a bulk c-plane AlN single crystal. The upper and lower insets are optical photo image (with each grid size of 1 × 1 mm^2^) and low-magnification SEM image (with a scale bar of 500 μm) of the bulk crystal, respectively.

Hexagonal AlN single crystals can be grown on the W substrate by inverse temperature-gradient method in the PVT process. The prepared crystals are evaluated by X-ray Diffraction (XRD) with Cu Kα radiation on a Philips X-ray diffractometer at 40 kV and 40 mA. A strong diffraction peak at 36.06° can be obtained which corresponds to the (002) lattice plane in parallel with the substrate, indicating the c-axis growth direction of the crystal. Rocking curves were taken in different areas on the (002) facet with a beam diameter of around 0.1mm and full-width at half maximum (FWHM) values of <70 arcsec were obtained. Figure 4 presents only one narrow peak with a FWHM close to 66 arcsec, showing a high crystalline quality of the typical c-plane bulk AlN single crystal. A small tail following the left side of curve indicates the probable existence of edge dislocation. The upper inset in Figure 4 is an optical photo image of the AlN sample exhibiting a yellow (amber) color. The yellow coloration is ascribed to optical absorption at around 2.7~2.9 eV, which is related to the aluminum vacancy (V_Al_)^3−/2−^ states and the presence of oxygen impurities [35,36,37]. The lower inset in Figure 4 is a low-magnification SEM image of the AlN sample with a scale bar of 500 μm, revealing excellent hexagonal morphology of the bulk c-plane crystal. In addition, the FWHM of around 144 arcsec for the (110) rocking curve is also obtained.

**Figure 5:**
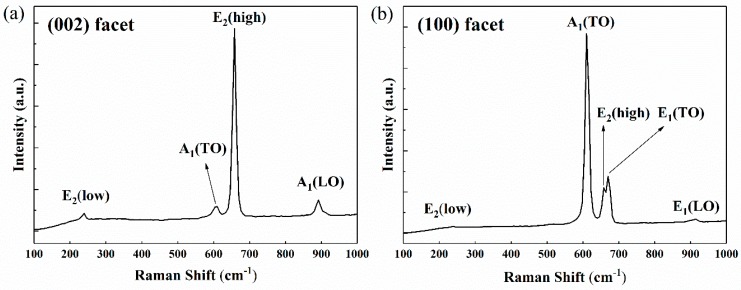
Raman spectra taken on (002) facet (**a**) and (100) facet (**b**) of the prepared bulk AlN single crystal at room temperature (300 K).

In order to further evaluate the crystalline quality, the prepared bulk AlN crystals were analyzed by Raman spectroscopy. Micro-Raman experiments were performed at room temperature (300 K) using a Raman Spectrometer (sourced from Zolix, Beijing, China) with 785nm laser as excitation source in backscattering geometry. Spectra were taken on c-facet and m-facet of the AlN single crystals under nearly normal incidence. Figure 5 shows the Raman spectrum obtained on (002) facet and (100) facet. For (002) facet, the E_2_(low) mode at 239 cm^−1^, the A_1_(TO) mode at 610 cm^−1^, the E_2_(high) mode at 658 cm^−1^ and the A_1_(LO) mode at 892 cm^−1^ are allowed while the E_1_(TO) mode and the E_1_(LO) mode are forbidden. For (100) facet, the E_2_(low) mode at 239 cm^−1^, the A_1_(TO) mode at 610 cm^−1^, the E_2_(high) mode at 658 cm^−1^, the E_1_(TO) mode at 670 cm^−1^ and the E_1_(LO) mode at 914 cm^−1^ are observed but the A_1_(LO) mode is not allowed. It should be clear that the phonon energies of TO and LO modes are influenced by different crystallographic facets, which is consistent with the result in ref [38]. The Raman phonon energies (frequencies) of all the observed modes are in good agreement with the results reported for nearly unstrained AlN [39,40] (see Table 2). The FWHMs of the E_2_(high) modes are 11 cm^−1^ and 12 cm^−1^, respectively.

Variations of Raman phonon energies between these AlN samples can be largely attributed to the stress caused by thermal expansion and lattice mismatch. The stress from lattice mismatch can be relieved by the formation of defects such as threading dislocations during growth. Due to the high growth temperature and the difference of thermal expansion coefficient between AlN and W, stress is introduced into both AlN crystals and W substrate during cooling. The frequency shifts of the E_2_(high) mode and the E_1_(TO) mode have been used to analyze the stress [41,42]. There exists a Raman stress factor (k) to link the shift of frequency (Δω) with the biaxial stress (σ), which can be described by the equation [41]

(3) Δω=kσ

As k is −6.3 ± 1.4 cm^−1^/GPa for E_2_(high) phonon, and Δω is approximately 1 cm^−1^ (the E_2_(high) phonon energy is 657.4 cm^−1^ ± 0.2 cm^−1^ for unstressed AlN), a compressive stress of 0.16 ± 0.04 GPa can be obtained using Equation (3) for the hexagonal AlN sample. Meanwhile, the E_1_(TO) phonon energy of the sample reveals a slight blueshift compared to the unstressed bulk AlN grown by spontaneous nucleation [42], which also reflects the slight compressive stress at the growing surface. The slight stress and narrow FWHM of mode phonon energy for E_2_(high) indicates a high crystalline quality of the AlN sample. The thickness of the AlN samples is around 2 mm. Note that the stress can be reduced by increasing thickness, thus the further improvement of crystalline quality can be realized in AlN-seeded growth.

## 5. Conclusions

For the past several decades, the PVT technology has been developed for AlN bulk growth. However, the harsh growth conditions, especially the narrow growth-temperature window, provide challenges for preparing high-quality AlN single crystals with a diameter of ≥2 inch. To explore the technology improvement of AlN bulk growth, three modified self-seeding PVT growth methods are discussed.

In comparison to the selected growth and separate freestanding growth, the inverse temperature-gradient method shows more advantages in terms of the diameter enlargement and quality improvement of bulk AlN single crystals as well as the process complexity. The inverse temperature-gradient method is able to reduce the nucleation rate and realize the single dominant growth on the crucible lid. Centimeter-sized bulk AlN single crystals used for seeded growth can be obtained by spontaneous nucleation using this method. X-ray rocking curve and Raman spectroscopy show high crystalline quality of the prepared AlN crystals. The inverse temperature-gradient method reveals a promising potential for the preparation of large-sized and high-quality AlN seed crystals used for seeded growth by virtue of its high efficiency, flexibility and cost-effectiveness.

## Figures and Tables

**Table 1:** Comparison of three strategies for AlN bulk growth.

Method	Selected Growth	Separate Freestanding Growth	Inverse Temperature-Gradient Growth
Crucible material	W	TaC	W
Nucleation position	Conical tip	Perforated sheet	Planar crucible lid
Key element	Nucleation site	Supersaturation	Temperature gradient
Advantages	Conical zone for dominant growth	Low nucleation rate, N-polar growth	Single dominant growth, relative low cost
Disadvantages	Complicated process	Carbon contamination, high manufacturing cost	High requirement of thermometry

**Table 2:** Raman phonon energies (cm^−1^) of the prepared AlN at room temperature.

Phonon Symmetry	Raman Phonon Energy (cm^−1^) for (002) Facet ^a^	Raman Phonon Energy (cm^−1^) for (100) Facet ^a^	Raman Phonon Energy (cm^−1^) ^b^	Raman Phonon Energy (cm^−1^) ^c^
E_2_(low)	239	239	249	249
A_1_(TO)	610	610	611	610
E_2_(high)	658	658	657	656
E_1_(TO)	-	670	671	669
A_1_(LO)	892	-	890	891
E_1_(LO)	-	914	912	912

^a^ This work. ^b^ Ref.39 AlN grown on α-Al_2_O_3_ by chloride-hydride–vapor-phase epitaxy (CHVPE). ^c^ Ref.40 AlN grown by a direct reaction of aluminum vapor with nitrogen.
